# Leiomyoma of the Trachea: a case report

**DOI:** 10.1186/s13019-015-0283-0

**Published:** 2015-05-31

**Authors:** Masahiro Kitada, Shunsuke Yasuda, Kei Ishibashi, Satoshi Hayashi, Yoshinari Matuda, Yoshinobu Ohsaki, Naoyuki Miyokawa

**Affiliations:** 1 Department of Respiratory Center, Asahikawa Medical University, Midorigaoka-Higashi 2-1-1-1, Asahikawa, Hokkaido 078-8510 Japan; 2 Department of Clinical Pathology, Asahikawa Medical University, Midorigaoka-Higashi 2-1-1-1, Asahikawa, Japan

**Keywords:** Tracheal tumor, Leiomyoma, Tracheoplasty

## Abstract

We present a surgical case of a rare primary tracheal tumor. In a 44-year-old asymptomatic man, computed tomography (CT), performed as part of health check-up, revealed a tumor measuring 1.5 cm in diameter in the mediastinal trachea. Biopsy failed to yield a definitive diagnosis, but the tumor tended to grow rapidly; therefore, surgery was performed. Five tracheal rings were resected through median sternotomy, followed by interrupted suture with 3–0 absorbable thread. The postoperative course has been favorable with no evidence of recurrence. The pathological diagnosis was leiomyoma. We report this case with literature review.

## Background

Primary tracheal tumor is a relatively rare condition. More than 90 % of all cases of primary tracheal tumor are malignant, and benign leiomyomas account for approximately 1 % of such tumors [1–3]. When a tumor forms in the trachea, it grows and thereby causes airway obstruction symptoms such as asphyxia. Therefore, treatment needs to be initiated immediately after detection. We present a surgical case of primary tracheal tumor that was detected through health check-up.

## Case presentation

**Fig. 1:**
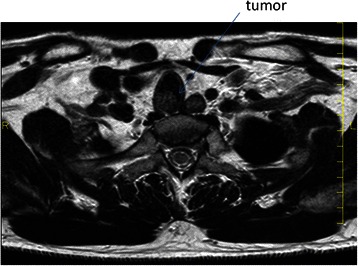
MRI showed a smooth, well-circumscribed nodular shadow was revealed in the posterior wall of the thoracic trachea. The arrow shows a neoplasm in the trachea

**Fig. 2:**
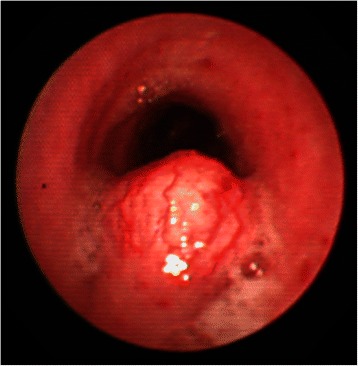
Bronchoscopy revealed a smooth submucosal tumor with abundant neovessels

**Fig. 3:**
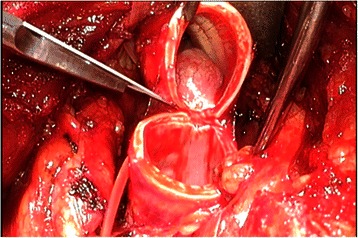
Operative findings (1). The tumor was occurred from membranous portion of trachea

**Fig. 4:**
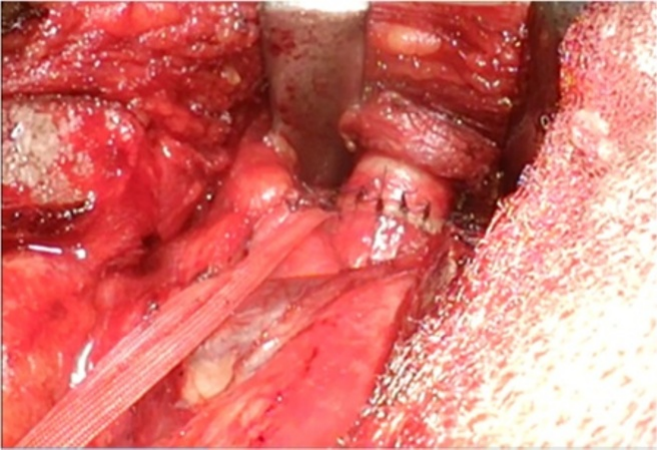
Operative findings (2). End-to-end anastomosis of the trachea was performed using a 3–0 monofilament synthetic absorbable suture

**Fig. 5:**
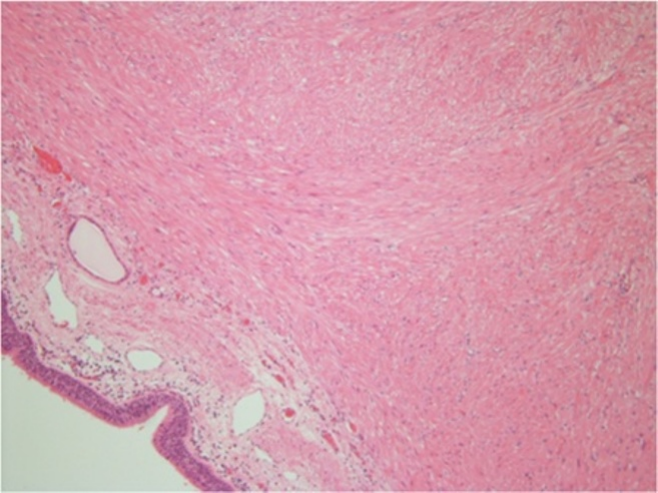
Histopathological examination revealed hyperplasia of smooth muscle cells that appeared neither mixed nor atypical, was noted beneath the mucous membrane. (HE × 40)

**Fig. 6:**
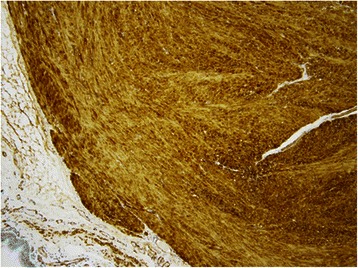
Immunohistochemical examination revealed for α-smooth muscle actin (×100)

The patient was a 44-year-old man with a timorous lesion in the trachea detected on computed tomography (CT) screening for lung cancer. He was referred to our hospital for further examination and treatment. No particular subjective symptoms were observed. The patient was 171 cm tall, weighed 73 kg, and had clear respiratory sounds. There was no palpable enlargement of surface lymph nodes. There were no abnormalities in his blood biochemistry findings, and tumor markers were all within the normal ranges. Spirometry results were normal (Vital Capacity: 3590 ml, forced expiratory volume in 1 s: 3310 ml). On CT and magnetic resonance imaging (MRI), a smooth, well-circumscribed nodular shadow measuring 1.3 × 1.5 cm was revealed in the posterior wall of the thoracic trachea 5 cm below the glottis (Fig. 1). Bronchoscopy revealed a smooth submucosal tumor with abundant neovessels (Fig. 2). Both brush cytology and forceps biopsy failed to yield a definitive diagnosis. Although no extratracheal involvement was noted, the tumor grew in a short time, and the possibility of malignancy could not be ruled out. Therefore, the decision was made to remove the tumor via tracheal segmental resection. The surgery was performed through median sternotomy. The thymus was removed, and the surrounding structures were separated to expose the trachea. Five tracheal rings, including the tumor, were removed by sleeve resection. During the surgery, the patient’s airway was secured by intubation. The intraoperative pathological diagnosis of the tumor was leiomyoma. Without any additional resection, end-to-end anastomosis of the trachea was performed using a 3–0 monofilament synthetic absorbable suture. The suture site was covered using the thymus (Figs. 3 and 4). The postoperative course was favorable. To date, there has been no evidence of recurrence or other problems for 1 year after surgery. The resected specimen was a yellow-whitish tumor measuring 20 mm in size localized in the tracheal lumen. Pathologically, hyperplasia of smooth muscle cells that appeared neither mixed nor atypical, was noted beneath the mucous membrane. Immunostaining for α-smooth muscle actin (α-SMA) was positive, leading to a diagnosis of benign leiomyoma (Figs. 5 and 6). The postoperative course has been favorable with no evidence of recurrence to date.

## Discussion

Primary tracheal tumor is a relatively rare disease. Of all cases of primary tracheal tumor, more than 90 % are malignant tumors, such as adenoid cystic carcinoma and squamous cell carcinoma, and tracheal leiomyomas are said to account for approximately 1 % of such lesions [1–3]. Additionally, leiomyomas occurring in the respiratory tract are reported to account for approximately 2 % of all cases of benign lung tumors [4]. Depending on the anatomical site of occurrence, leiomyomas of the respiratory tract can be classified as pulmonary parenchymal or tracheobronchial type. Pulmonary leiomyomas are asymptomatic, and they are often detected via routine health check-up. A preoperative diagnosis is therefore difficult to make, and surgical resection of the tumor is often required to establish a diagnosis. There are also cases of pulmonary metastasizing leiomyoma originating from uterine fibroids [5]. On the contrary, when a tracheobronchial leiomyoma is detected, it is rarely asymptomatic, unlike in this case, and patients often present with coughing, breathing difficulty, bloody sputum, and bronchitis prior to being diagnosed. When a leiomyoma occurs in the trachea in particular, enlargement of the tumor can lead to breathing difficulty and possibly asphyxia depending on the tumor progression. This is why treatment needs to be initiated immediately after detection. To make a definitive diagnosis of a tracheal tumor, bronchoscopic biopsy can be used. However, as it is a submucosal tumor and there is a potential risk for airway obstruction caused by bleeding, bronchoscopy is difficult to perform in some cases, which may require an intraoperative pathological diagnosis.

Removal of the tumor is the basic treatment, but the question is whether to take a surgical or endoscopic approach. In recent years, some reports have been published on resected cases of relatively small, pedunculated tumors in which a high frequency snare [3, 6] or Yttrium Aluminum Garnet (YAG) laser [7, 8] was used. In broad-based tumors, however, these methods can potentially result in bleeding, rupture of the membranous portion, or residual tumor, and therefore, surgical resection is often selected. There is a report describing that surgery for a pulmonary leiomyoma often includes pneumectomy [9]. Although some patients with primary tracheal leiomyomas reportedly undergo tumor removal through tracheotomy [10], the surgical procedure usually consists of tracheal segmental resection and direct anastomosis [11]. To ligate the trachea, an absorbable polypropylene suture is reportedly used in many cases. Unlike malignant tumors, extensive resection is not required, and thus, mobilization of the trachea and other associated procedures are not performed.

## Conclusion

We reported a case of primary tracheal leiomyoma, a rare disease, in which we performed tracheal segmental resection and anastomosis.

## Consent

Written informed consent was obtained from the patients for publication of this case report and any accompanying images. A copy of the written consent is available for review by the Editor-in Chief of this journal.
